# An Analysis of the Incidence and Cost of Intracranial Aneurysm and Subarachnoid Haemorrhage Treatment between 2013 and 2021

**DOI:** 10.3390/ijerph20053828

**Published:** 2023-02-21

**Authors:** Andrzej Śliwczyński, Maciej Jewczak, Małgorzata Dorobek, Kamila Furlepa, Izabela Gołębiak, Edyta Skibińska, Iwona Sarzyńska-Długosz

**Affiliations:** 1 Faculty of Medicine, Lazarski University, 02-662 Warsaw, Poland; 2 Department of Operations Research, Faculty of Economics and Sociology, University of Lodz, 90-214 Lodz, Poland; 3 Central Clinical Hospital of the Ministry of Interior and Administration, 02-507 Warsaw, Poland; 4 Hospital and Healthcare Management, College of Business Administration, American University in the Emirates, Dubai P.O. Box 503000, United Arab Emirates; 5 2nd Department of Neurology, Institute of Psychiatry and Neurology, 02-957 Warsaw, Poland

**Keywords:** unruptured intracranial aneurysms, subarachnoid haemorrhage, incidence, cost of treatment

## Abstract

The incidence of unruptured intracranial aneurysms (UIAs) amounts to 3.2% among adults. The annual risk of aneurysm rupture is 2–10% and it results in subarachnoid haemorrhage (SAH). The aim of this study is to assess changes in the incidence of unruptured intracranial aneurysms and subarachnoid haemorrhages in Poland between 2013 and 2021 and the cost associated with their in-hospital treatment in the acute phase. The analysis was based on the National Health Fund database. Patients diagnosed with UIA and SAH and hospitalised between 2013 and 2021 were chosen. The statistical analysis was performed with an assumed significance level of α = 0.05. The ratio between the prevalence of SAH and UIA diagnoses was 4:6. The proportion of women in relation to men was higher in both diagnoses. The highest proportions of patients with diagnoses SAH and UIA were found in highly urbanised provinces. The value of medical services in 2021 compared to 2013 increased by 81.8%. The highest values in this period were recorded in Mazowieckie province, and the lowest were recorded in Opolskie province. The overall number of patients hospitalised with diagnosis of UIA or SAH did not decrease, but the risk of aneurysm rupture probably decreased, which resulted in lower incidence of SAH in subsequent years of observation. The recorded changes in the dynamics of the value of medical services per patient or per hospitalisation largely coincided. However, it is difficult to speculate on expected value levels as not all provinces showed linear changes in the value of services provided.

## 1. Introduction

The prevalence of unruptured intracranial aneurysms (UIAs), which are pathological dilatations of the main branching cerebral vessels, is estimated to be 3.2% in the adult population. It is 1.6 times higher among women but does not differ between European, Japanese or North American populations [1,2]. Due to the increasing availability and quality of neuroimaging examinations, intracranial aneurysms are detected with increasing frequency, often completely incidentally during imaging examinations not directly related to aneurysm symptoms, e.g., during the diagnosis of headaches or dizziness or during imaging in the course of other diseases such as stroke, trauma to the head area, psychiatric disorders or cancer. After the detection of a UIA on neuroimaging, the aneurysmal dilatation of the vessel may remain asymptomatic and stable and non-progressive during the follow-up, or a systematic increase in the aneurysm dimension may be observed or rupture may occur due to a gradual increase in the size of the aneurysm. However, UIA rupture can also occur without a concomitant increase in aneurysm size. The annual risk of aneurysm rupture depends on the size, shape and location of the aneurysm and ranges from 2% to 10% [3]. The consequence of aneurysm rupture is subarachnoid haemorrhage (SAH), which despite advances in risk assessment, imaging, surgical and procedural techniques and intensive care, is a subtype of stroke with a poor prognosis [2]. Due to the frequent rupture of aneurysms at a young age, SAH significantly reduces productive life years [4], has a high mortality rate (between 35% and 45%) and a high percentage of motor and/or cognitive disability among those who survive [5]. Up to 76% of patients who survive a subarachnoid haemorrhage from a ruptured aneurysm have persistent cognitive deficits and remain dependent on others, and only 6–17% return to work [6]. The rate of potential life years lost due to SAH is similar to the rate of life years lost due to ischaemic stroke and haemorrhagic stroke [7].

Based on the results of prospective cohort and case-control studies, the 5-year risk of untreated aneurysm rupture has been estimated to range from 0.4% to 17.8% [8,9]. The overall incidence of SAH is 9.1 per 100,000 person-years in most regions of the world, yielding approximately 36,000 SAH cases per year in Europe [10]. The incidence of SAH increases with the age of the patient, the mean age of the first SAH incidence in a lifetime is 52 years [11], whereas the median incidence of first SAH ranges from 50 to 60 years [12,13,14].

As the majority of patients diagnosed with unruptured intracranial aneurysms are at a relatively young age, with a life expectancy well beyond 5 years [15,16,17], it makes sense to introduce therapeutic approaches aimed at reducing the risk of UIA rupture and SAH occurrence [10]. 

Prevention of UIA rupture by endovascular or neurosurgical treatment can reduce the risk of SAH, but both treatments carry the risk of serious complications. The European Stroke Organisation (ESO) recommendations of 2013 emphasised that the larger the UIA, the more likely it is to rupture, but the decision on the indication for its closure should be made individually by a multidisciplinary team by considering the risk of spontaneous rupture, the surgical risk and the expected benefit (life expectancy with little or no deficit) [10]. There are certain groups of patients in whom therapeutic decisions are particularly difficult. This group includes, for example, patients with multiple UIAs after SAH. The results of lately published long-term follow-up study evaluating the risk of rupture of UIAs in such patients can be helpful in decision making and suggest that patients with a moderate or severe disability after SAH have a relatively low risk of rupture of UIAs as well as the higher treatment risks [18].

The aim of our study was to assess changes in the incidence of hospitalisations with the main diagnosis of unruptured intracranial aneurysms or subarachnoid haemorrhages in Poland between 2013 and 2021 and the costs associated with their treatment in the acute phase.

## 2. Materials and Methods

The database of the National Health Fund (NFZ) of the national payer of healthcare services in Poland that registers every publicly funded medical event was analysed. The following parameters indicated by neurologists were selected for extracting and aggregating data:

- Years from 2013 to 2021;
- Patients hospitalised with the main diagnosis of unruptured intracranial aneurysm (UIA);
- Patients hospitalised with the main diagnosis of subarachnoid haemorrhage (SAH) regardless of its aetiology.

Statistical inference was performed at the assumed significance level of α = 0.05. 

Cost values were obtained from a state institution, the only public payer in Poland—the National Health Fund. The payer receives from medical entities a list of all performed medical procedures with accuracy for each patient, for which he then pays according to rules specified in the law and in the agreement between the medical facility and the National Health Fund. For the purpose of the article, at the request of the authors, the payer summed up all the costs of procedures performed for the patient during treatment concerning only the procedures discussed in the article.

Additional materials, in particular values of medical services converted from the local currency (PLN) according to current prices to values based on purchasing power parity for the year 2021 with the assumption that USD 1 PPP = PLN 1.837, published by the OECD [19], were attached to the study (Appendix A), allowing easier comparison of analyses performed in other countries.

## 3. Results

The number of patients reported by healthcare providers with SAH or UIA diagnoses averages approximately 7800 patients per year. The proportion between identified SAH diagnoses and those of UIA is a 4 to 6 ratio (Table 1).

In the group of patients with identified SAH, the proportion of women was greater than men, averaging approximately 59% per year. In the group of patients with UIA, the proportion of women was also significantly higher than men and amounted to approximately 73% on average per year. The dynamics of the number of patients counted per year with SAH diagnosis showed a decreasing trend at an average annual level of approximately (−3.51%) the largest decreases in the number of patients were observed in 2016 (−9.19%) and 2018 (−9.60%). The rate of change in the number of patients year on year shown with diagnosis UIA indicated a clear upward trend averaging approx. “+3%” subject to high variability. The value of the lowest dynamics occurred in 2020 and the highest in 2014 and 2021 (Table 2).

When analysing the frequency of reporting a given type of diagnosis in total, it can be seen that since 2014, there has been a reversal in the frequency of diagnoses with a predominance of UIA diagnoses. While in the case of SAH diagnoses, one can speak of a decreasing significant linear trend indicating a year-on-year decrease by an average of 121 patients, in the case of UIA diagnoses, the trend is more towards an power form with a partial elasticity of 0.1392%.


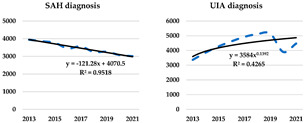


The pattern of identified tendencies by diagnosis confirms the significance of the trend reversal between SAH and UIA diagnoses. It is also apparent from the course of the patient distribution that the effects of the COVID pandemic (or perhaps more the lockdown policy and restrictions on health service provision) have more severely impacted the detection of UIA cases. The downward trend identified in 2019–2020 is returning to the previous pre-pandemic rate in 2021. In cases of diagnosed SAHs, virtually no impact of the pandemic is evident—in this group of cases, and the downward trend is preserved throughout the time sample.

The distribution of the number of patients with reported SAH and UIA diagnoses shows great variability across the provinces. The highest values were reported in highly urbanised provinces (i.e., Dolnoslaskie, Mazowieckie, Slaskie, Wielkopolska), whose total annual mean proportion of patients was 48.50% (Table A1 in Appendix A).

The provinces with the highest mean annual number of patients with SAH diagnosis are the following: Mazowieckie (approx. 488), Slaskie (approx. 388), Wielkopolskie (approx. 351) and Malopolskie (approx. 330). After standardising the results on the basis of the number of inhabitants of a given province, the provinces with the highest rate of the number of patients per 100 thousand are Swietokrzyskie, 10.21; Podkarpackie, 10.19; Wielkopolskie, 10.08. The lowest mean annual number of patients with this diagnosis was recorded in the following provinces: Lubuskie (approx 75), Podlaskie (approx. 90), Opolskie (approx. 94) and Warmińsko-Mazurskie (approx. 123). The incidence rate per 100 000 inhabitants was lowest in the following provinces: Lubelskie (6.58), Lubuskie (7.46) and Podlaskie (7.60). The mean population-standardised incidence rate registered for Poland was 9.80.

Regarding the patients with UIA, the provinces with the highest number of patients were Mazowieckie (approx. 705), Slaskie (approx. 550), Wielkopolskie (approx. 543) and Dolnoslaskie (approx. 511). After standardising the results on the basis of the number of inhabitants of a given province, the regions with the highest rate of patients per 100,000 inhabitants consisted of Dolnoslaskie (17.63), Podlaskie (16.40), Lubelskie (15.94). The lowest number was observed in the following provinces: Lubuskie (approx. 44), Opolskie (approx. 60), Warminsko-Mazurskie (approx. 85) and Swietokrzyskie (approx. 130). The incidence rate per 100,000 inhabitants was lowest in the following provinces: Lubuskie (4.32), Warminsko-Mazurskie (5.93) and Opolskie (6.07). The mean population-*standardised* incidence rate registered in Poland was 9.81. There is a scatter of index values (SD = ±3.19) for UIA diagnosis.

**Figure 1:**
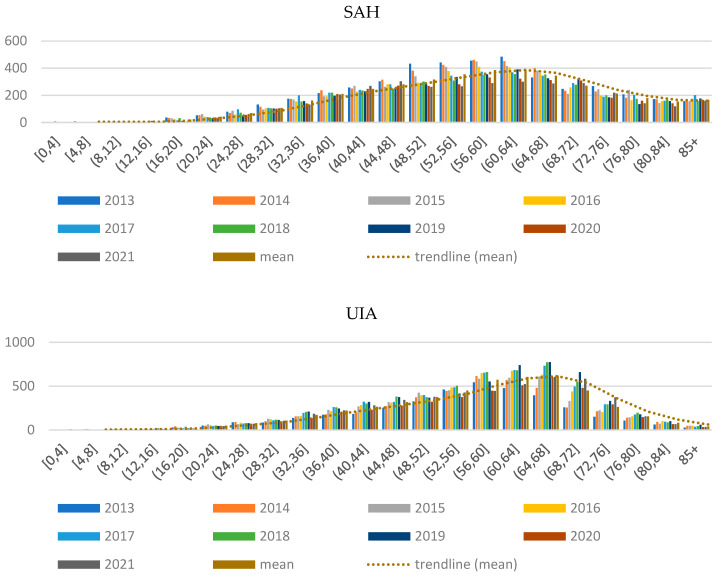
The distribution of the number of patients according to age.

Age-related vascular degradation has a significant impact on the frequency of patients with the diagnoses *analysed*. The number of patients according to their age is presented (Figure 1).

The median number of patients reported with SAH diagnosis on average annually of over 200 patients is in the age range between 36 and 72 years. The dominant of medical events represent patients aged 60–64 years. The median number of patients reported with UIA diagnosis is in the same age range of 36–72 years. In contrast, the dominant age for medical events is between 64 and 68 years.

### The Analysis of Public Financing and Settlement of Therapies

In 2013, the provinces with the highest value of registered medical services were Wielkopolskie, with a value of more than PLN 48 million; Mazowieckie (more than PLN 38 million); and Slaskie and Dolnoslaskie, with values exceeding PLN 22 million. Compared to the year 2013, there was an 81.8% increase in the value of services in 2021. In this period, and the highest values were recorded in the Mazowieckie province with a level of over PLN 75 million. The Wielkopolskie province recorded the highest value of medical services at the level of PLN 71 million. The province which recorded the highest average annual dynamics of change was Swietokrzyskie, for which the rate of change amounted to +35.7% per year. It should be noted, however, that this was the region with one of the lowest shares of the total value of benefits (3% in 2013). An increase in the value of medical services per patient between 2013 and 2021 occurred in the Lubelskie (+170.01%), Dolnoslaskie (+106.76%), Slaskie (+93.37%) and Swiętokrzyskie provinces (+142.75%). Within the UIA diagnosis group, a change in the distribution by province of medical services per patient is noted. The levels of medical services per patient recorded in 2013 virtually doubled in 2021. The increase in the variation is confirmed by the distribution coefficients, which amounted to 64.76% for SAH diagnoses. For UIA diagnoses, a concordance level of 83.75% was observed, which allows us to conclude that the changes in medical services values per patient between 2013 and 2021 were quite similar.

Opolskie province recorded the lowest levels of the value of medical services provided—the value of services decreased on average by 4.02% per year between 2013 and 2021, from PLN 3.7 million in 2013 to PLN 3.14 million in 2021. Opolskie province also showed the lowest levels of service value per patient in both extreme years of the analysis.

The highest increases in the value of benefits were recorded in Mazowieckie province, + PLN 1.61 million (average year-on-year increase of +13.48%); Dolnoslaskie, +PLN 1.57 million (average year-on-year increase by +16.26%); and Slaskie, +PLN 1.01 million (average year-on-year increase by +12.40%). The region with the highest average year-on-year dynamics was Lubuskie, with the rate of +35.17%, which, however, was only an increase in the value of services by PLN 147,000. The province with the highest value of medical services per patient treated in 2013 was Wielkopolskie, which was followed in 2021 by Dolnoslaskie. 

**Figure 2:**
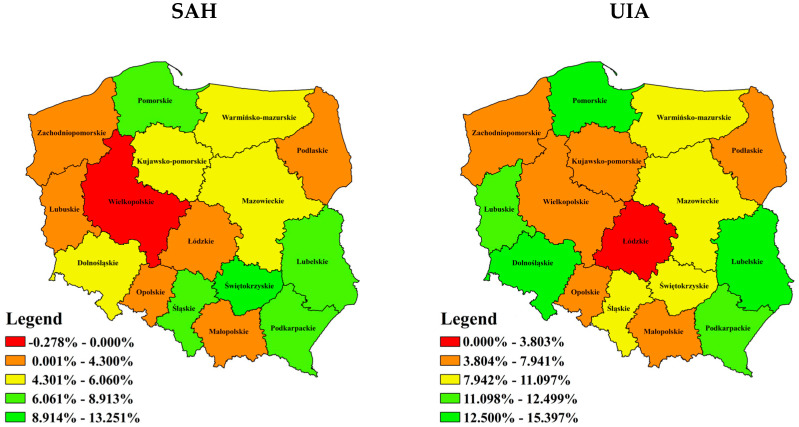
The dynamics of 2013–2021 change in value of medical services paid per patient.

The average annual dynamics of the value of patient expenditure incurred between 2013 and 2021 was calculated with a distinction between diagnoses (Figure 2).

What is an interesting finding is the negative average annual rate of change recorded for Wielkopolskie province in diagnosis SAH group—this was the only province showing a negative rate of change. According to expenditure per patient, Swietokrzyskie province recorded the highest average annual rate of change between 2013 and 2021. Changes in the level of expenses on diagnosis UIA group were characterised by higher average annual dynamics in the period 2013–2021, with Pomorskie, Lubelskie and Dolnoslaskie provinces being in the group of objects with the highest variability.

It is indisputable that there has been an increase in the value of services provided per patient over the analysed 8 years. This is shown both by the absolute value of the cost volume and the average annual change rates. However, the increases in the value of funds paid by the national payer (NHF) for medical services provided in relation to therapy did not show a linear course in all provinces, which makes the management of treatment costs as well as detection and prevention procedures more complex.

In order to adequately identify the changes that occurred between 2013 and 2021 on the cost side of aneurysm treatment by diagnosis group, models of the development tendencies and medium-term rate of change were estimated for each of the provinces in Poland (Appendix B). Thanks to the multivariate search, it was possible through the evaluation of the constants of the time models to assess the initial level of expenditure in each of the spatial objects (Table 3). 

By identifying the parameters of the time variable, it is possible to identify the level of average year-on-year increase in historical cost levels, and it is also possible to project this change for future periods. It follows from the indications (excluding statistically insignificant directional coefficients from this interpretation) that both at the macro level for Poland and at the level of the provinces, increases in expenditure per patient were recorded. Regions with non-linear course of value growth of medical services include, for SAH diagnoses, Lubuskie, Lodzkie, Opolskie, Podlaskie, Wielkopolskie and Zachodniopomorskie provinces. However, for UIA diagnoses only in Lodzkie province, changes in the value of medical services per patient did not occur linearly. What is a rather interesting conclusion drawn from the regression analyses is the relationship between the changes in the parameters of the time *t* variable between the diagnoses SAH and UIA groups. For the majority of provinces, the average change for UIA diagnosis were much more significant than the increase in expenditure per patient in the SAH group, for instance the extreme levels of difference in levels in this relationship was recorded in Lubuskie 51.67 for SAH versus 1007.65 for UIA diagnosis. However, for Lodzkie, Podkarpackie and Swietokrzyskie, this relation was reverse, while for the Slaskie province the average increase recorded in 2013–2021 was practically in the ratio of 1 to 1.

The concordance in the directional coefficients of the linear models for SAH and UIA diagnoses (after rejecting models with insignificant changes over time) was 98%, which confirms similar quantitative changes in the increase in the value of services per patient; however, in the case of medium-term dynamics in percentage terms, the concordance drops to 44%. At the same time, a higher mean increasing dynamics for UIA diagnoses compared to SAH is noted. The only province which recorded negative dynamics of average annual change in the years 2013–2021 was Wielkopolskie, whose value of services per patient with SAH diagnosis decreased on average by 0.56% each year. Similar relationships and trends can be observed for the value of services per hospitalisation.

The periods from 2016 to 2017 and from 2017 to 2018 showed the most dynamic changes (Table 4), with the former mainly recording decreases in the value of services per patient, except for Opolskie and Lubuskie provinces. In the latter period, there was a complete reversal of the tendencies, when practically only increases in the value of services per patient were recorded.

## 4. Discussion

Analysing the incidence of subarachnoid haemorrhages according to gender, we find that in the Polish population they occur about 1.4-fold more frequently among women than men, while unruptured intracranial aneurysms occur about 2.7-fold more frequently among women than men. This is a much greater gender difference in the incidence of UIA than has been published to date. This is probably due to the much older study population. In the analysed group, the highest number of patients reported with a diagnosis of UIA was aged 60–64 years, but even after taking into account the age distribution of the study population, this difference is significant [12]. It is interesting to note the systematic decrease in the number of reported subarachnoid haemorrhages with a concomitant increase in the number of reported unruptured intracranial aneurysms. An undoubted factor contributing to the increasing number of diagnosed aneurysms is the progress in diagnosis and the increased availability of neuroimaging examinations throughout Poland. This is confirmed by the fact that the highest number of UIA patients is reported in the provinces with the highest urbanisation rates. In turn, the decrease in the number of reported subarachnoid haemorrhages, in our opinion, may be due to the dissemination of prophylactic treatment of UIA after the publication of the ESO guidelines indicating the rationale for individual decision-making regarding such treatment after taking into account the risk of aneurysm rupture, the potential benefits of aneurysm closure as well as perioperative risk [10]. In estimating the potential risk of SAH due to intracranial aneurysm rupture, the following should be considered: patient demographic characteristics (such as age, gender, ethnic race), family history of intracranial aneurysms, previous episode of subarachnoid haemorrhage, risk factors for aneurysm rupture (i.e., hypertension, smoking) and aneurysm characteristics (such allocation, size, shape and number of aneurysms, dynamics of aneurysm enlargement) [20,21,22,23,24,25,26,27,28,29]. Additionally, a predictive model such as the PHASES score can be used in estimating the risk of aneurysm rupture [8]. A similar trend of an increase in hospitalisations associated with prophylactic treatment of unruptured intracranial aneurysms is reported in the literature [30,31].

Progressive changes in the value of services provided under diagnoses groups: SAH and UIA show a strong upward trend, with more dynamic changes identified in the group of UIA diagnoses. Overall for Poland between 2013 and 2021, the mean increase in the value of services per patient per year for SAH diagnoses was PLN 10,624.72, and for UIA diagnoses it was PLN 19,382.25.

The Lodzkie province was the only one to show no linear increase in the value of services per patient in the UIA diagnosis group, while in the SAH diagnosis group the following provinces can be indicated: Lubuskie, Lodzkie, Podlaskie, Wielkopolskie and Zachodniopomorskie. At the same time, Lubuskie province noted a decrease of PLN 2,321.76 in the value of services per patient in the SAH group, while there was a PLN 5,072.28 increase in the value of services per patient in the UIA group. Swietokrzyskie province is also worth mentioning, which noted very similar changes in the SAH and UIA diagnosis groups. Analysing the coefficients of the trend function, on average each year there was an increase in the value of services per patient in the SAH group by PLN 1 591.81 (which indicated a yearly increase of 28.26%), while in the UIA diagnosis group one can indicate an increase in the value of services per patient by PLN 1 585.99 PLN (yearly increase of 20.11%). This region recorded both the highest average annual change in PLN and percentage change in the SAH diagnosis group. Although the highest mean value of services per patient was recorded between 2013 and 2021 in Dolnoslaskie province for UIA diagnoses (an increase of PLN 2,126.34 from period to period), Pomorskie province recorded the highest average annual value growth of 33.16% (indicating an annual increase of PLN 1831.63).

The barrier of our study is limitation of the analysis, i.e., pursuant to the Public Statistics Act and the provisions of RODO; in order to keep sensitive data belonging to patients anonymous, the analyses were performed on aggregated data.

The limitation of our study is the diagnosis of SAH. This diagnosis includes all cases of SAH regardless of their aetiology. Indeed, it should be noted that in approximately 15% of non-traumatic SAHs by angiography, we do not find the source of the bleeding. The majority of these are pericerebral haemorrhages with a milder course and better long-term prognosis [10]. In our study, these were included along with all cases of SAH. 

A limitation of our study may also be the assessment of the number of unruptured intracranial aneurysms reported without an analysis that includes personal data to check the number of services reported for a given patient during both a calendar year and subsequent years. According to the guidelines, patients undergoing aneurysm closure treatment should be radiologically monitored in order to assess the effectiveness of the treatment applied as well as to detect possible aneurysm growth [32,33,34], particularly as the percentage of patients undergoing additional follow-up treatment ranges from 5% to 20% after endovascular aneurysm closure and from 3% to 9% after surgical treatment [34].

As a consequence of the identified relationships, it can be concluded that for the purposes of planning future levels of service provision in SAH and UIA diagnosis groups, it is more difficult to predict the levels of expenses incurred or the number of patients served in subsequent periods, when these changes do not progress linearly. This problem may also be compounded considerably by the occurrence of abnormal events, such as a pandemic, which studies show significantly alters the structure of funding streams or the number of health services provided.

Preventive treatment of aneurysms has consistently increased its usefulness for health compared with conservative treatment [35]. In order to assess the usefulness of preventive management in terms of the cost-effectiveness of such treatment in Poland, the next step in our study should be to analyse the costs associated with surgical treatment compared with those associated with endovascular treatment, especially as it has been proven that the medical costs associated with the care of the patient with a ruptured intracranial aneurysm are different in different regions of the world. In the United States, the total cost of hospital treatment and annual medical care are the same for both surgical and endovascular treatment, whereas in countries such as South Korea and China, endovascular procedures were costlier [36]. Conducting such analyses should also be done to support national health policy and decision-making, although when analysing the cost of care of patients after subarachnoid haemorrhage, it should be borne in mind that treatment in the acute phase is only part of the costs generated by patients who suffer subarachnoid haemorrhage. The total care expenditures must also include the cost of long-term rehabilitation and care of the patient with disabilities as a result of SAH.

## 5. Conclusions

1. The overall number of patients hospitalised with diagnosis of UIA or SAH is not decreasing in subsequent years of observation. Thanks to early diagnosis and proper treatment of UIA, the risk of aneurysm rupture is probably declining, which results in improving the overall population health by lowering incidence of SAH—the type of stroke of relatively poor prognosis.
2. The changes that were observed in the rate of change of the value of services prescribed per patient or per hospitalisation were highly consistent.
3. When the gender of the patient was taken into account, the degree of concordance between the rate of change and the overall rate, as measured by the Spearman rank correlation coefficient, was 98% for women and 78% for men. When comparing the SAH and UIA, the convergence rates were 84% and 80%, respectively.
4. Not all provinces showed linear changes in the value of services provided, which unfortunately makes it difficult to speculate on their expected levels in subsequent periods of analysis. It is also worth remembering the recorded high inflation in the economy and paying attention to the dynamics of realised values of SAH and UIA diagnoses in constant prices according to the purchasing power parity of USD PPPs, which shows that in the years 2013–2021 in most of the provinces the increases did not exceed 10% of the completed services (Appendix A).

## Figures and Tables

**Table 1:** The number of patients with an identified diagnosis by year.

Diagnosis/Sex	2013	2014	2015	2016	2017	2018	2019	2020	2021
**SAHall**	3944	3857	3765	3448	3563	3251	3262	3075	3012
Women	2316	2270	2185	2019	2147	1906	1921	1843	1766
Men	1628	1587	1580	1429	1416	1345	1341	1232	1246
**UIAall**	3366	3858	4276	4621	4925	5112	5145	3901	4471
Women	2462	2744	3073	3328	3572	3737	3799	2883	3342
Men	904	1114	1203	1293	1353	1375	1346	1018	1129

**Table 2:** The rate of patients with an identified diagnosis by year per 100,000 population.

Diagnosis/Sex	2013	2014	2015	2016	2017	2018	2019	2020	2021
**SAH all**	10.25	10.02	9.80	8.97	9.27	8.46	8.50	8.04	7.91
Women	11.66	11.43	11.01	10.18	10.82	9.61	9.69	9.33	8.98
Men	8.74	8.52	8.50	7.69	7.62	7.24	7.22	6.66	6.77
**UIA all**	8.74	10.03	11.12	12.02	12.81	13.31	13.40	10.19	11.74
Women	12.39	13.82	15.49	16.77	18.00	18.85	19.17	14.59	16.99
Men	4.85	5.98	6.47	6.95	7.28	7.40	7.25	5.50	6.13

**Table 3:** The change in levels of reported value of medical services per patient—a summary of linear trend functions and medium-term rates of change from 2013 to 2021 for SAH and UIA diagnoses *.

Description	Summary of Trend Models, with *p*-Values for Coefficient with Determination Factor, Standard Error of the Model and Average Annual Rate of Change Value of Services per Patient in PLN	
	SAH	UIA
Poland	$\begin{array}{c} \hat{C}=175,332+10,624.7t\\ \;\;\;\;\;\;\;\left(0.0001\right)\;\;\;\;\;\left(0.0061\right)\\ R^{2}=68.19\%\;\;S_{e}=21,241.98 \end{array}$ $\bar{i_{g}}$ = 10.75%	$\begin{array}{c} \hat{C}=114,296+19,382.2t\\ \;\;\;\;\;\;\;\left(0.0015\right)\;\;\;\;\;\left(0.0020\right)\\ R^{2}=76.63\%\;\;S_{e}=31,333.86 \end{array}$ $\bar{i_{g}}$ = 19.81%
Dolnoslaskie	$\begin{array}{c} \hat{C}=13,515.3+1021.97t\\ \;\;\;\;\;\;\;\left(0.0001\right)\;\;\;\;\;\left(0.009\right)\\ R^{2}=64.6\%\;\;\;S_{e}=2217.25 \end{array}$ $\bar{i_{g}}$ = 9.83%	$\begin{array}{c} \hat{C}=6752.14+2126.34t\\ \;\;\;\;\;\;\;\left(0.0333\right)\;\;\;\;\;\left(0.0022\right)\\ R^{2}=75.81\%\;\;S_{e}=3516.23 \end{array}$ $\bar{i_{g}}$ = 28.86%
Kujawsko-Pomorskie	$\begin{array}{c} \hat{C}=9711.93+717.952t\\ \;\;\;\;\;\;\;\left(0.0001\right)\;\;\;\;\;\left(0.0057\right)\\ R^{2}=68.76\%\;\;\;S_{e}=1416.58 \end{array}$ $\bar{i_{g}}$ = 12.32%	$\begin{array}{c} \hat{C}=9517.01+948.78t\\ \;\;\;\;\;\;\;\left(0.0005\right)\;\;\;\;\;\left(0.0104\right)\\ R^{2}=63.24\%\;\;S_{e}=2117.79 \end{array}$ $\bar{i_{g}}$ = 13.52%
Lubelskie	$\begin{array}{c} \hat{C}=4602.16+553.50t\\ \;\;\;\;\;\;\;\left(0.0001\right)\;\;\;\;\;\left(0.001\right)\\ R^{2}=80.51\%\;\;\;S_{e}=797.34 \end{array}$ $\bar{i_{g}}$ = 18.62%	$\begin{array}{c} \hat{C}=4820.54+1684.50t\\ \;\;\;\;\;\;\;\left(0.0411\right)\;\;\;\;\;\left(0.0017\right)\\ R^{2}=77.52\%\;\;\;S_{e}=2655.56 \end{array}$ $\bar{i_{g}}$ = 28.83%
Lubuskie	$\begin{array}{c} \hat{C}=7318.18+51.67t\\ \;\;\;\;\;\;\;\;\;\;\left(0.009\right)\;\;\;\left(0.893\right)\\ R^{2}=0.27\%\;\;\;S_{e}=2869.63 \end{array}$ $\bar{i_{g}}$ = 8.10%	$\begin{array}{c} \hat{C}=4546.33+1007.65t\\ \;\;\;\;\;\;\;\;\;\;\left(0.0113\right)\;\;\;\left(0.0038\right)\\ R^{2}=72.09\%\;\;\;S_{e}=1835.20 \end{array}$ $\bar{i_{g}}$ = 26.21%
Lodzkie	$\begin{array}{c} \hat{C}=11,366.5+517.35t\\ \;\;\;\;\;\;\;\;\;\;\left(0.001\right)\;\;\;\left(0.219\right)\\ R^{2}=20.61\%\;\;\;S_{e}=2971.88 \end{array}$ $\bar{i_{g}}$ = 7.81%	$\begin{array}{c} \hat{C}=7342.19+203.46t\\ \;\;\;\;\;\;\;\;\;\;\left(0.001\right)\;\;\;\left(0.3305\right)\\ R^{2}=13.51\%\;\;\;S_{e}=1507.20 \end{array}$ $\bar{i_{g}}$ = 7.75%
Malopolskie	$\begin{array}{c} \hat{C}=16,985.8+110.46t\\ \;\;\;\;\;\;\;\;\;\;\left(0.0001\right)\;\;\;\left(0.788\right)\\ R^{2}=11.0\%\;\;\;S_{e}=3065.36 \end{array}$ $\bar{i_{g}}$ = 7.12%	$\begin{array}{c} \hat{C}=8653.87+830.49t\\ \;\;\;\;\;\;\;\;\;\;\left(0.0005\right)\;\;\;\left(0.0136\right)\\ R^{2}=60.48\%\;\;\;S_{e}=3065.36 \end{array}$ $\bar{i_{g}}$ = 14.74%
Mazowieckie	$\begin{array}{c} \hat{C}=11,429.0+832.37t\\ \;\;\;\;\;\;\;\;\;\;\left(0.0001\right)\;\;\;\left(0.002\right)\\ R^{2}=74.95\%\;\;\;S_{e}=1408.50 \end{array}$ $\bar{i_{g}}$ = 10.71%	$\begin{array}{c} \hat{C}=9120.13+1918.22t\\ \;\;\;\;\;\;\;\;\;\;\left(0.0007\right)\;\;\;\left(0.0003\right)\\ R^{2}=86.80\%\;\;\;S_{e}=2189.85 \end{array}$ $\bar{i_{g}}$ = 21.86%
Opolskie	$\begin{array}{c} \hat{C}=5330.12+353.26t\\ \;\;\;\;\;\;\;\;\;\;\left(0.0008\right)\;\;\;\left(0.073\right)\\ R^{2}=38.83\%\;\;\;S_{e}=1297.93 \end{array}$ $\bar{i_{g}}$ = 5.72%	$\begin{array}{c} \hat{C}=3614.71+505.18t\\ \;\;\;\;\;\;\;\;\;\;\left(0.0052\right)\;\;\;\left(0.0162\right)\\ R^{2}=58.59\%\;\;\;S_{e}=1243.37 \end{array}$ $\bar{i_{g}}$ = 13.36%
Podkarpackie	$\begin{array}{c} \hat{C}=8557.02+1101.70t\\ \;\;\;\;\;\;\;\left(0.0004\right)\;\;\;\left(0.0025\right)\\ R^{2}=75.22\%\;\;\;S_{e}=1850.87 \end{array}$ $\bar{i_{g}}$ = 15.96%	$\begin{array}{c} \hat{C}=3002.01+731.32t\\ \;\;\;\;\;\;\;\left(0.0123\right)\;\;\;\left(0.0025\right)\\ R^{2}=75.01\%\;\;\;S_{e}=1235.62 \end{array}$ $\bar{i_{g}}$ = 26.56%
Podlaskie	$\begin{array}{c} \hat{C}=14,018.0+360.31t\\ \;\;\;\;\;\;\;\left(0.0004\right)\;\;\;\left(0.2198\right)\\ R^{2}=20.60\%\;\;\;S_{e}=2070.97 \end{array}$ $\bar{i_{g}}$ = 8.79%	$\begin{array}{c} \hat{C}=6891.95+551.43t\\ \;\;\;\;\;\;\;\left(0.0004\right)\;\;\;\left(0.0248\right)\\ R^{2}=53.66\%\;\;\;S_{e}=1500.01 \end{array}$ $\bar{i_{g}}$ = 12.12%
Pomorskie	$\begin{array}{c} \hat{C}=8556.29+766.64t\\ \;\;\;\;\;\;\;\left(0.0017\right)\;\;\;\left(0.0417\right)\\ R^{2}=46.93\%\;\;\;S_{e}=2386.44 \end{array}$ $\bar{i_{g}}$ = 14.10%	$\begin{array}{c} \hat{C}=3501.33+1831.63t\\ \;\;\;\;\;\;\;\left(0.0588\right)\;\;\;\left(0.0003\right)\\ R^{2}=86.29\%\;\;\;S_{e}=2137.14 \end{array}$ $\bar{i_{g}}$ = 33.16%
Slaskie	$\begin{array}{c} \hat{C}=10,321.7+1121.65t\\ \;\;\;\;\;\;\;\left(0.0001\right)\;\;\;\left(0.0025\right)\\ R^{2}=75.22\%\;\;\;S_{e}=1884.50 \end{array}$ $\bar{i_{g}}$ = 15.84%	$\begin{array}{c} \hat{C}=7215.75+1237.08t\\ \;\;\;\;\;\;\;\left(0.0112\right)\;\;\;\left(0.0132\right)\\ R^{2}=60.79\%\;\;\;S_{e}=2908.60 \end{array}$ $\bar{i_{g}}$ = 20.94%
Swietokrzyskie	$\begin{array}{c} \hat{C}=5878.66+1591.84t\\ \;\;\;\;\;\;\;\left(0.0499\right)\;\;\;\left(0.0087\right)\\ R^{2}=64.99\%\;\;\;S_{e}=3419.94 \end{array}$ $\bar{i_{g}}$ = 28.26%	$\begin{array}{c} \hat{C}=6780.06+1585.99t\\ \;\;\;\;\;\;\;\left(0.0217\right)\;\;\;\left(0.0061\right)\\ R^{2}=68.16\%\;\;\;S_{e}=3173.08 \end{array}$ $\bar{i_{g}}$ = 20.11%
Warminsko-Mazurskie	$\begin{array}{c} \hat{C}=10,441.5+741.65t\\ \;\;\;\;\;\;\;\left(0.0002\right)\;\;\;\left(0.0288\right)\\ R^{2}=51.78\%\;\;\;S_{e}=2095.13 \end{array}$ $\bar{i_{g}}$ = 12.49%	$\begin{array}{c} \hat{C}=3319.80+1385.45t\\ \;\;\;\;\;\;\;\left(0.1698\right)\;\;\;\left(0.0088\right)\\ R^{2}=64.84\%\;\;\;S_{e}=2986.31 \end{array}$ $\bar{i_{g}}$ = 23.43%
Wielkopolskie	$\begin{array}{c} \hat{C}=21,889.1+140.123t\\ \;\;\;\;\;\;\;\left(0.0001\right)\;\;\;\left(0.6803\right)\\ R^{2}=02.57\%\;\;\;S_{e}=2525.35 \end{array}$ $\bar{i_{g}}$ = −0.56%	$\begin{array}{c} \hat{C}=16,085.4+1330.40t\\ \;\;\;\;\;\;\;\left(0.0005\right)\;\;\;\left(0.0261\right)\\ R^{2}=53.01\%\;\;\;S_{e}=3666.99 \end{array}$ $\bar{i_{g}}$ = 13.40%
Zachodniopomorskie	$\begin{array}{c} \hat{C}=15,410.2+642.24t\\ \;\;\;\;\;\;\;\left(0.0001\right)\;\;\;\left(0.0725\right)\\ R^{2}=38.94\%\;\;\;S_{e}=2354.50 \end{array}$ $\bar{i_{g}}$ = 6.49%	$\begin{array}{c} \hat{C}=13,133.2+1504.29t\\ \;\;\;\;\;\;\;\left(0.0010\right)\;\;\;\left(\;0.0100\right)\\ R^{2}=63.58\%\;\;\;S_{e}=3333.13 \end{array}$ $\bar{i_{g}}$ = 16.51%

* The empirical significance level for the parameter is given in brackets, while red colour indicates parameters that are not statistically significant of the directional coefficients of the development trend function at the assumed significance level. Recorded medium-term annual rates $\bar{i_{g}}$ of change also confirm a low coefficient of determination of the temporal linear models.

**Table 4:** The change in levels of reported services per patient by value for provinces from 2013 to 2021 in total and for SAH and UIA diagnoses.

Description	2014/2013		2015/2014		2016/2015		2017/2016		2018/2017		2019/2018		2020/2019		2021/2020	
	SAH	UIA	SAH	UIA	SAH	UIA	SAH	UIA	SAH	UIA	SAH	UIA	SAH	UIA	SAH	UIA
Poland	662.49	658.49	792.15	381.68	359.55	820.65	−2855.35	−3253.87	3410.57	5749.92	958.19	1143.84	1433.86	2387.88	1628.54	4091.34
Dolnoslaskie	−607.78	1868.88	1441.59	725.07	1731.64	2791.63	−3678.35	−6347.08	4652.07	8435.16	−1411.17	1509.68	6801.34	4933.16	−1643.21	5226.23
Kujawsko-pomorskie	153.09	1322.21	2112.44	−895.98	1305.52	642.75	−3044.70	−3175.73	914.61	4617.72	3798.48	1747.46	−356.03	2604.70	1383.71	936.31
Lubelskie	675.69	1248.23	336.73	244.91	727.63	−1155.99	−68.68	−24.46	−695.00	5635.70	2736.19	1446.58	−492.05	1356.72	2014.27	6534.43
Lubuskie	−428.04	1056.71	−1911.80	1378.18	−619.92	−2668.46	−2321.76	5072.28	2545.17	−1083.05	−2231.03	−1116.97	2605.58	4542.98	5845.19	2240.10
Lodzkie	−856.73	−411.03	4103.27	365.25	−5024.54	1434.32	−833.73	−3974.94	2704.11	2180.59	−1942.88	−315.08	7949.94	1598.48	−1475.36	1994.08
Malopolskie	1950.92	−506.31	827.11	3083.26	−226.68	1758.89	−7562.30	−5662.37	5394.84	4838.94	800.29	851.72	−2268.44	1092.18	6356.44	1841.43
Mazowieckie	854.29	1527.38	1053.15	468.75	1092.69	1522.24	−3008.01	−2219.11	4404.88	6182.99	−327.39	1465.97	3150.81	4488.63	−939.70	1937.30
Opolskie	−226.61	509.58	−1645.35	−662.14	1827.29	−1081.55	436.28	1447.88	2082.32	740.25	804.15	3281.70	−2845.62	−1606.65	1167.54	617.98
Podkarpackie	1235.92	647.02	532.13	738.88	2416.54	−594.21	−3397.48	−236.01	7681.66	926.32	−1401.96	1544.67	777.52	1544.78	15797.00	2462.16
Podlaskie	3871.29	1188.37	1365.85	1218.27	361.21	−752.11	−5096.83	−2733.48	5048.43	4955.26	−80.52	−500.63	−867.32	1781.11	150.43	−901.50
Pomorskie	−1119.84	875.79	528.77	1145.95	−671.84	364.43	−96.56	85.05	2399.62	7257.84	−1885.12	−3021.49	3680.32	5279.16	5249.17	2975.91
Slaskie	−222.27	1187.53	1619.53	−739.38	393.11	852.22	−1683.04	−4093.66	2377.47	6502.86	1160.69	301.80	2934.78	2874.96	3844.79	5011.84
Swietokrzyskie	585.72	−735.59	2450.50	−552.44	−885.45	1969.79	−2687.75	−4167.73	2431.40	6632.96	5859.09	3658.13	−53.58	858.73	8547.43	5163.61
Warminsko-mazurskie	−570.33	339.59	1891.63	880.30	237.78	−1074.14	−1691.19	−615.59	−1454.64	1674.90	4745.06	4433.13	954.01	6892.53	3368.45	−3366.31
Wielkopolskie	2015.17	852.50	−3271.76	66.23	1158.88	314.28	−3975.47	−5547.11	6844.71	7880.84	1277.66	4482.01	−3074.55	−3235.55	−1462.61	8023.80
Zachodniopomorskie	4669.65	381.23	−3893.70	1323.72	1795.98	458.95	−13.84	−4914.72	−1582.00	5500.44	6197.20	4805.89	823.68	−223.39	−3658.48	6947.40

Note: for better identification, positive changes (increasing) are marked in green, while negative changes (decreasing) are marked in red.

**Figure A1:**
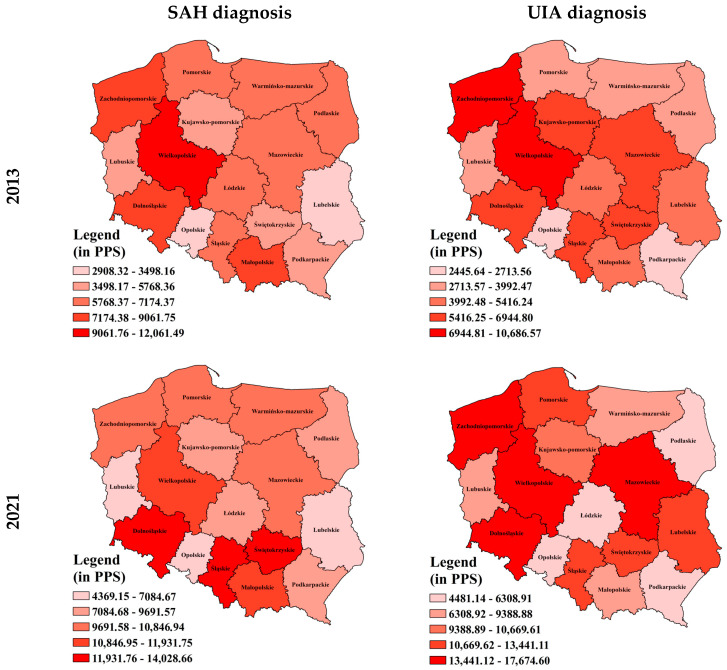

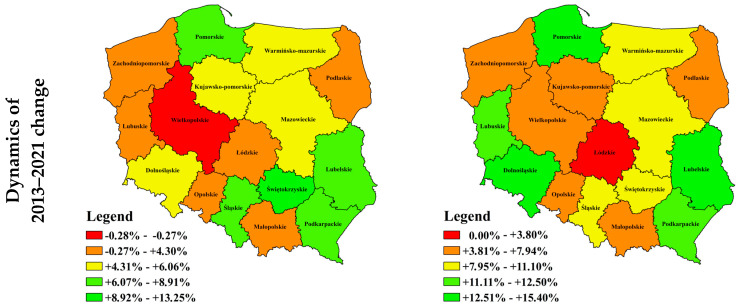
The value of services paid per patient in 2013 and 2021.

## Data Availability

The data presented in this study are available on request from the corresponding author.

## Appendix A

**Table A1:** The rate of patients with the type of the diagnosis reported by province per 100,000 inhabitants.

Province/Diagnosis	2013	2014	2015	2016	2017	2018	2019	2020	2021
**Dolnoslaskie**	24.36	25.13	23.93	25.9	29.22	32.47	32.48	25.32	26.21
SAH	11.55	11.62	10.67	8.85	9.61	8.44	8.97	7.68	8.96
UIA	12.82	13.51	13.26	17.05	19.6	24.02	23.52	17.64	17.25
**Kujawsko-Pomorskie**	19.88	21.96	25.21	23.13	24.48	21.9	26.01	19.5	22.07
SAH	9.75	10.62	11.41	9.84	8.98	6.98	8.69	7.81	7.03
UIA	10.13	11.34	13.8	13.29	15.51	14.92	17.32	11.69	15.04
**Lubelskie**	21.01	22.02	26.22	24.75	23.94	24.74	25.52	17.28	17.19
SAH	8.21	8.05	8.18	7.03	6.16	6.09	5.45	5.3	4.72
UIA	12.8	13.97	18.04	17.72	17.78	18.65	20.06	11.98	12.47
**Lubuskie**	11.16	12.55	10.9	11.01	12.1	10.94	11.17	11.02	15.21
SAH	9.79	9.51	6.68	7.08	6.79	6.01	6.43	6.45	8.41
UIA	1.37	3.04	4.22	3.93	5.31	4.93	4.74	4.57	6.81
**Lodzkie**	17.31	19.29	18.89	19.19	19.71	18.69	18.9	14.89	14.07
SAH	11.3	11.58	9.3	9.46	10.62	9.73	8.6	8.16	7.53
UIA	6.01	7.71	9.58	9.74	9.09	8.96	10.31	6.73	6.54
**Malopolskie**	15.71	17.43	18.12	18.63	21.76	20.17	18	17.15	17.61
SAH	9.94	11.67	10.94	10.05	10.79	9.38	8.36	8.27	8.45
UIA	5.77	5.76	7.18	8.57	10.97	10.79	9.65	8.88	9.16
**Mazowieckie**	21.29	21.28	22.41	23.65	24.27	23.26	23.66	18.56	21.35
SAH	10.61	9.52	10.38	9.04	9.16	8.38	8.78	7.98	7.93
UIA	10.68	11.75	12.04	14.61	15.12	14.88	14.88	10.58	13.41
**Opolskie**	16.93	17.09	14.06	16.01	19.09	18.55	18.01	11.67	8.56
SAH	12.84	10.59	7.43	9.06	11.92	11.35	9.16	7.47	5.47
UIA	4.08	6.49	6.63	6.95	7.17	7.2	8.85	4.2	3.09
**Podkarpackie**	17.71	17.05	16.69	18	19.26	17.66	18.66	17.87	19.8
SAH	11.79	10.14	10.86	10.34	10.85	10	10.15	8.25	9.33
UIA	5.92	6.9	5.83	7.66	8.41	7.66	8.51	9.62	10.47
**Podlaskie**	21.26	26.68	29.1	26.88	26.76	25.31	23.93	16.11	20
SAH	9.04	8.39	9.08	7.08	7.18	6.77	7.89	5.28	7.72
UIA	12.22	18.29	20.02	19.8	19.59	18.54	16.04	10.82	12.27
**Pomorskie**	13.59	14.68	14.56	14.64	15.14	15.26	15.19	14.06	15.81
SAH	9.36	8.99	8.84	8.29	8.43	7.8	8.49	8.05	8.39
UIA	4.23	5.69	5.72	6.35	6.71	7.46	6.7	6.01	7.41
**Slaskie**	18.42	18.75	21.09	19.7	20.82	22.63	24.81	20.57	19.44
SAH	9.41	9.05	9.74	8.16	8.27	7.81	8.32	8.64	7.61
UIA	9	9.7	11.35	11.54	12.55	14.82	16.49	11.93	11.83
**Swietokrzyskie**	16.24	19.87	24.82	22.03	21.96	18.93	17.91	20.09	24.16
SAH	10.88	10.61	9.78	10.62	11.22	10.63	8.59	10.7	8.82
UIA	5.36	9.26	15.03	11.41	10.74	8.3	9.32	9.39	15.34
**Warminsko-Mazurskie**	10.09	14.2	12.92	13.92	15.41	14.98	15.74	17.51	15.87
SAH	6.63	9.42	7.92	7.8	9.14	9.17	8.29	10.02	8.89
UIA	3.46	4.78	5	6.13	6.28	5.81	7.45	7.48	6.97
**Wielkopolskie**	26.65	26.29	26.59	27.4	27.37	27.59	23.52	19.79	25.97
SAH	12.14	10.65	10.65	10.68	10.12	9.56	9.43	9.01	8.51
UIA	14.51	15.64	15.94	16.72	17.25	18.03	14.09	10.78	17.45
**Zachodniopomorskie**	16.7	17.49	17.77	17.04	17.59	16.11	18.45	17.3	16.52
SAH	8.96	8.86	8.71	7.55	8.5	7.11	8.37	7.52	7.33
UIA	7.74	8.63	9.06	9.48	9.09	8.99	10.08	9.77	9.18

Value of services paid per patient in PPP value terms (USD PPP) from the year 2021.

**Table A2:** Changes in the levels of reported services per patient by value in PLN (PLN) for provinces in the years 2013–2021 in total and for SAH and UIA diagnoses.

Description	2014/2013		2015/2014		2016/2015		2017/2016		2018/2017		2019/2018		2020/2019		2021/2020	
	SAH	UIA	SAH	UIA	SAH	UIA	SAH	UIA	SAH	UIA	SAH	UIA	SAH	UIA	SAH	UIA
Poland	360.63	358.46	431.22	207.77	195.73	446.73	−1554.35	−1771.3	1856.6	3130.06	521.61	622.67	780.54	1299.88	886.52	2227.19
Dolnoslaskie	−330.86	1017.36	784.75	394.7	942.65	1519.67	−2002.37	−3455.13	2532.43	4591.81	−768.19	821.82	3702.42	2685.44	−894.51	2844.98
Kujawsko-pomorskie	83.34	719.76	1149.94	−487.74	710.68	349.89	−1657.43	−1728.76	497.88	2513.73	2067.76	951.26	−193.81	1417.91	753.24	509.69
Lubelskie	367.82	679.49	183.3	133.32	396.1	−629.28	−37.39	−13.31	−378.33	3067.88	1489.49	787.47	−267.85	738.55	1096.5	3557.12
Lubuskie	−233.01	575.24	−1040.72	750.23	−337.46	−1452.62	−1263.89	2761.17	1385.5	−589.58	−1214.5	−608.04	1418.39	2473.04	3181.92	1219.43
Lodzkie	−466.38	−223.75	2233.68	198.83	−2735.19	780.79	−453.86	−2163.82	1472.03	1187.04	−1057.64	−171.52	4327.68	870.16	−803.13	1085.51
Malopolskie	1062.01	−275.62	450.25	1678.42	−123.4	957.48	−4116.66	−3082.4	2936.77	2634.15	435.65	463.65	−1234.86	594.55	3460.23	1002.41
Mazowieckie	465.04	831.45	573.3	255.17	594.83	828.66	−1637.46	−1208.01	2397.87	3365.81	−178.22	798.02	1715.2	2443.46	−511.54	1054.6
Opolskie	−123.36	277.4	−895.67	−360.44	994.71	−588.76	237.49	788.18	1133.54	402.96	437.75	1786.44	−1549.06	−874.61	635.57	336.41
Podkarpackie	672.79	352.22	289.67	402.22	1315.48	−323.47	−1849.47	−128.47	4181.63	504.26	−763.18	840.87	423.26	840.92	2.41	1340.32
Podlaskie	2107.4	646.91	743.52	663.18	196.63	−409.42	−2774.54	−1488.02	2748.19	2697.47	−43.83	−272.52	−472.14	969.57	81.89	−490.75
Pomorskie	−609.61	476.75	287.84	623.82	−365.73	198.38	−52.57	46.3	1306.27	3950.92	−1026.19	−1644.79	2003.44	2873.8	2857.47	1619.99
Slaskie	−121	646.45	881.62	−402.49	213.99	463.92	−916.19	−2228.45	1294.21	3539.93	631.84	164.29	1597.6	1565.03	2092.97	2728.27
Swietokrzyskie	318.85	−400.43	1333.97	−300.73	−482.01	1072.29	−1463.12	−2268.77	1323.57	3610.75	3189.49	1991.36	−29.17	467.46	4652.93	2810.89
Warminsko-mazurskie	−310.47	184.86	1029.74	479.2	129.44	−584.72	−920.63	−335.1	−791.86	911.76	2583.05	2413.25	519.33	3752.06	1833.67	−1832.5
Wielkopolskie	1096.99	464.07	−1781.03	36.05	630.86	171.08	−2164.11	−3019.65	3726.02	4290.06	695.51	2439.85	−1673.68	−1761.32	−796.2	4367.88
Zachodniopomorskie	2542	207.53	−2119.6	720.59	977.67	249.84	−7.53	−2675.4	−861.18	2994.25	3373.54	2616.16	448.38	−121.6	−1991.55	3781.93

Note: for better identification, positive changes (increasing) are marked in green, while negative changes (decreasing) are marked in red.

**Table A3:** Changes in levels of reported value of services per patient in PPP value terms (USD PPP) by province from 2013 to 2021 in total and for SAH and UIA diagnoses.

Description	2014/2013		2015/2014		2016/2015		2017/2016		2018/2017		2019/2018		2020/2019		2021/2020	
	SAH	UIA	SAH	UIA	SAH	UIA	SAH	UIA	SAH	UIA	SAH	UIA	SAH	UIA	SAH	UIA
Poland	196.31	195.13	234.74	113.10	106.55	243.18	−846.14	−964.24	1010.67	1703.90	283.95	338.96	424.90	707.61	482.59	1212.41
Dolnoslaskie	−180.11	553.82	427.19	214.86	513.15	827.26	−1090.02	−1880.85	1378.57	2499.62	−418.18	447.37	2015.47	1461.86	−486.94	1548.71
Kujawsko-pomorskie	45.37	391.81	625.99	−265.51	386.87	190.47	−902.25	−941.08	271.03	1368.39	1125.62	517.83	−105.50	771.86	410.04	277.46
Lubelskie	200.23	369.89	99.78	72.57	215.62	−342.56	−20.35	−7.25	−205.95	1670.05	810.83	428.67	−145.81	402.04	596.90	1936.37
Lubuskie	−126.84	313.14	−566.53	408.40	−183.70	−790.76	−688.02	1503.09	754.22	−320.95	−661.13	−331.00	772.12	1346.24	1732.13	663.82
Lodzkie	−253.88	−121.80	1215.94	108.24	−1488.94	425.04	−247.07	−1177.91	801.32	646.18	−575.74	−93.37	2355.84	473.69	−437.20	590.91
Malopolskie	578.12	−150.04	245.10	913.67	−67.17	521.22	−2240.97	−1677.95	1598.68	1433.94	237.15	252.40	−672.22	323.65	1883.63	545.68
Mazowieckie	253.15	452.61	312.08	138.91	323.81	451.09	−891.38	−657.60	1305.32	1832.23	−97.02	434.41	933.70	1330.14	−278.46	574.09
Opolskie	−67.15	151.01	−487.57	−196.21	541.49	−320.50	129.28	429.06	617.06	219.36	238.30	972.48	−843.26	−476.11	345.98	183.13
Podkarpackie	366.24	191.74	157.69	218.95	716.10	−176.09	−1006.79	−69.93	2276.34	274.50	−415.45	457.74	230.41	457.77	1.31	729.62
Podlaskie	1147.20	352.16	404.75	361.01	107.04	−222.87	−1510.36	−810.03	1496.02	1468.41	−23.86	−148.35	−257.02	527.80	44.58	−267.15
Pomorskie	−331.85	259.53	156.69	339.59	−199.09	107.99	−28.62	25.20	711.09	2150.75	−558.62	−895.37	1090.60	1564.40	1555.51	881.87
Slaskie	−65.87	351.91	479.92	−219.10	116.49	252.54	−498.74	−1213.09	704.52	1927.02	343.95	89.43	869.68	851.95	1139.34	1485.18
Swietokrzyskie	173.57	−217.98	726.17	−163.71	−262.39	583.72	−796.47	−1235.04	720.51	1965.57	1736.25	1084.03	−15.88	254.47	2532.90	1530.15
Warminsko-mazurskie	−169.01	100.63	560.56	260.86	70.46	−318.30	−501.16	−182.42	−431.06	496.33	1406.12	1313.69	282.71	2042.49	998.19	−997.55
Wielkopolskie	597.16	252.62	−969.53	19.62	343.42	93.13	−1178.07	−1643.79	2028.32	2335.36	378.61	1328.17	−911.09	−958.80	−433.42	2377.72
Zachodniopomorskie	1383.78	112.97	−1153.84	392.26	532.21	136.00	−4.10	−1456.40	−468.80	1629.97	1836.44	1424.15	244.08	−66.19	−1084.13	2058.75

Note: for better identification, positive changes (increasing) are marked in green, while negative changes (decreasing) are marked in red.

## Appendix B. Statistical Methods Used in Paper

In order to check the value of health benefits of changes (in terms of absolute change), the values of absolute increments were estimated over time, comparing pairs of consecutive time periods at a time:

$$\Delta_{i}=y_{t}-y_{t-1}$$

where *t* is the current period of time, *t* − 1 is the period 1 unit of time earlier.

To assess a longer tendencies of average changes, the geometric mean was used (the *T* degree root of the quotient of the absolute magnitudes of the phenomenon under study in the last period/moment over the first one). The average rate of change of the phenomena included in the form of a time series was calculated using a single-basis approach, according to the formula:

$${\bar{l}}_{g}=\sqrt[{T}]{\frac{y_{t}}{y_{1}}}$$

where: *T* indicates the length of elapsed years/periods of time.

The medium-term rate of change informs about the average volatility of the phenomenon every period/moment under the assumption that the changes are unidirectional over the entire sample span. The use of the geometric mean allowed to estimate how the volatility of the analysed aggregates changed over the years 2013–2021, indicating the percentage change per 1 year.

As a measure of the convergence/consistency of the distributions of the variables at the chosen time points and also in examining the correlation between the variables to see if two quantitative variables are related the non-parametric Spearman’s rank coefficient was calculated as follows:

$$r_{xy}=1-\frac{6\sum d_{i}{}^{2}}{n^{3}-n}$$

where *d_i_* = (*R_Xi_* − *R_Yi_*) is the difference between the ranks of variables *X* and *Y* for *i*-th observation, *n* is the number of analysed observations. The higher the value of the Spearman’s rank coefficient module, the higher the degree of concordance the evaluated variables showed, while the sign of the non-parametric correlation coefficient indicated concordance or the complete opposite of the course of concordance.

For the linear time regression models of the form used:

$$y_{t}=\beta_{0}+\beta_{1}t+\xi$$

in which the estimated parameter *β*_1_ is a marginal propensity to consume that reports the change in the dependent variable caused by a unit change of time. The sign of the *β*_1_ parameter indicates an increase (positive sign) or decrease (negative sign) in the average level of the phenomenon over time. For the estimation of partial elasticities, a power model (non-linear model reduced to linear form) of the form was used:

$$y_{t}=\beta_{0}t^{\beta_{1}}e^{\xi }$$

The elasticities-parameter *β*_1_ made it possible to assess approximately how much the dependent variable would change if the independent variable changed by 1 % point.

The quality of fit of the regression models was verified with the usage of:

- The coefficient of determination *R*^2^, which indicates how much of the total variation in the dependent variable is explained by the mode and is calculated as follows:
  $$R^{2}=1-\frac{\sum {\left(y_{t}-\hat{y_{t}}\right)}^{2}}{\sum {\left(y_{t}-\bar{y_{t}}\right)}^{2}}$$
  where$\;y_{t}$ is the current value of dependent variable, $\hat{y_{t}}$ is the current value (theoretical) of the dependent variable obtained from the model and ${\bar{y}}_{t}$ is the average value of dependent variable;

- The standard error of the model which indicates how much the average observed values of the dependent variable differ ± from the theoretical values of this variable determined from the model and is calculated as follows:
  $$S_{e}=\sqrt{\frac{\sum {\left(y_{t}-\hat{y_{t}}\right)}^{2}}{n-k-1}}$$
  where n−k−1 is a level of degrees of freedom of the model.

## References

[1] Vlak M.H., Algra A., Brandenburg R., Rinkel G.J. (2011). Prevalence of unruptured intracranial aneurysms, with emphasis on sex, age, comorbidity, country, and time period: A systematic review and meta-analysis. Lancet Neurol..

[2] Lawton M.T., Vates G.E. (2017). Subarachnoid Hemorrhage. N. Engl. J. Med..

[3] van der Kamp L.T., Rinkel G.J.E., Verbaan D., Berg R.V.D., Vandertop W.P., Murayama Y., Ishibashi T., Lindgren A., Koivisto T., Teo M. (2021). Risk of rupture after intracranial aneurysm growth. JAMA Neurol..

[4] Schatlo B., Fung C., Stienen M.N., Fathi A.R., Fandino J., Smoll N.R., Zumofen D., Daniel R.T., Burkhardt J.-K., Bervini D. (2021). Incidence and Outcome of Aneurysmal Subarachnoid Hemorrhage: The Swiss Study on Subarachnoid Hemorrhage (Swiss SOS). Stroke.

[5] Nieuwkamp D.J., Setz L.E., Algra A., Linn F.H., de Rooij N.K., Rinkel G.J. (2009). Changes in case fatality of aneurysmal subarachnoid haemorrhage over time, according to age, sex, and region: A meta-analysis. Lancet Neurol..

[6] Al-Khindi T., MacDonald R.L., Schweizer T.A. (2010). Cognitive and functional outcome after aneurysmal subarachnoid hemorrhage. Stroke J. Cereb. Circ..

[7] Johnston S.C., Selvin S., Gress D.R. (1998). The burden, trends, and demographics of mortality from subarachnoid hemorrhage. Neurology.

[8] Greving J.P., Wermer M.J., Brown R.D., Morita A., Juvela S., Yonekura M., Ishibashi T., Torner J.C., Nakayama T., Rinkel G.J.E. (2014). Development of the PHASES score for prediction of risk of rupture of intracranial aneurysms: A pooled analysis of six prospective cohort studies. Lancet Neurol..

[9] Tominari S., Morita A., Ishibashi T., Yamazaki T., Takao H., Murayama Y., Sonobe M., Yonekura M., Saito N., Shiokawa Y. (2015). Prediction model for 3-year rupture risk of unruptured cerebral aneurysms in Japanese patients. Ann. Neurol..

[10] Steiner T., Juvela S., Unterberg A., Jung C., Forsting M., Rinkel G. (2013). European Stroke Organization Guidelines for the Management of Intracranial Aneurysms and Subarachnoid Haemorrhage. Cerebrovasc. Dis..

[11] Etminan N., Chang H.S., Hackenberg K., De Rooij N.K., Vergouwen M.D.I., Rinkel G.J.E., Algra A. (2019). Worldwide incidence of aneurysmal subarachnoid hemorrhage according to region, time period, blood pressure, and smoking prevalence in the population: A systematic review and meta-analysis. JAMA Neurol..

[12] de Rooij N.K., Linn F.H., van der Plas J.A., Algra A., Rinkel G.J. (2007). Incidence of subarachnoid haemorrhage: A systematic review with emphasis on region, age, gender and time trends. J. Neurol. Neurosurg. Psychiatry.

[13] Fogelholm R., Hernesniemi J., Vapalahti M. (1993). Impact of early surgery on outcome after aneurysmal subarachnoid hemorrhage. A population based study. Stroke.

[14] Rinkel G.J., Djibuti M., Algra A., van Gijn J. (1998). Prevalence and risk of rupture of intracranial aneurysms: A systematic review. Stroke.

[15] Etminan N., Rinkel G.J. (2016). Unruptured intracranial aneurysms: Development, rupture and preventive management. Nat. Rev. Neurol..

[16] MacDonald R.L., Schweizer T.A. (2017). Spontaneous subarachnoid haemorrhage. Lancet.

[17] Brown R.D., Broderick J.P. (2014). Unruptured intracranial aneurysms: Epidemiology, natural history, management options, and familial screening. Lancet Neurol..

[18] Juvela S. (2021). Outcome of Patients with Multiple Intracranial Aneurysms after Subarachnoid Hemorrhage and Future Risk of Rupture of Unruptured Aneurysm. J. Clin. Med..

[19] OECD Data. https://data.oecd.org/conversion/purchasing-power-parities-ppp.htm.

[20] Juvela S., Poussa K., Lehto H., Porras M. (2013). Natural history of unruptured intracranial aneurysms: A long-term follow-up study. Stroke.

[21] Wiebers D.O., Whisnant J.P., Huston J. (2003). Unruptured intracranial aneurysms: Natural history, clinical outcome, and risks of surgical and endovascular treatment. Lancet.

[22] Woo D., Khoury J., Haverbusch M.M., Sekar P., Flaherty M.L., Kleindorfer D.O., Kissela B.M., Moomaw C.J., Deka R., Broderick J.P. (2009). Smoking and family history and risk of aneurysmal subarachnoid hemorrhage. Neurology.

[23] Morita A., Kirino T., Hashi K., Aoki N., Fukuhara S., Hashimoto N., Nakayama T., Sakai M., Teramoto A., UCAS Japan Investigators (2012). The natural course of unruptured cerebral aneurysms in a Japanese cohort. N. Engl. J. Med..

[24] Etminan N., Beseoglu K., Steiger H.J., Hanggi D. (2011). The impact of hypertension and nicotine on the size of ruptured intracranial aneurysms. J. Neurol. Neurosurg. Psychiatry.

[25] Tada Y., Wada K., Shimada K., Makino H., Liang E.I., Murakami S., Kudo M., Kitazato K.T., Nagahiro S., Hashimoto T. (2014). Roles of hypertension in the rupture of intracranial aneurysms. Stroke J. Cereb. Circ..

[26] Bijlenga P., Ebeling C., Jaegersberg M., Summers P., Rogers A., Waterworth A., Iavindrasana J., Macho J., Pereira V.M., Bukovics P. (2013). Risk of rupture of small anterior communicating artery aneurysms is similar to posterior circulation aneurysms. Stroke.

[27] Bijlenga P., Gondar R., Schilling S., Morel S., Hirsch S., Cuony J., Corniola M.-V., Perren F., Rüfenacht D., Schaller K. (2017). PHASES Score for the Management of Intracranial Aneurysm: A Cross-Sectional Population-Based Retrospective Study. Stroke.

[28] Korja M., Lehto H., Juvela S. (2014). Lifelong rupture risk of intracranial aneurysms depends on risk factors: A prospective Finnish cohort study. Stroke J. Cereb. Circ..

[29] Wermer M.J., Greebe P., Algra A., Rinkel G.J. (2005). Incidence of recurrent subarachnoid hemorrhage after clipping for ruptured intracranial aneurysms. Stroke J. Cereb. Circ..

[30] Huang M.C., Baaj A.A., Downes K., Youssef A.S., Sauvageau E., van Loveren H.R., Agazzi S. (2011). Paradoxical trends in the management of unruptured cerebral aneurysms in the United States: Analysis of nationwide database over a 10-year period. Stroke.

[31] Silva N.A., Shao B., Sylvester M.J., Eloy J.A., Gandhi C.D. (2018). Unruptured aneurysms in the elderly: Perioperative outcomes and cost analysis of endovascular coiling and surgical clipping. Neurosurg. Focus.

[32] Thompson B.G., Brown R.D., Amin-Hanjani S., Broderick J.P., Cockroft K.M., Connolly E.S., Duckwiler G.R., Harris C.C., Howard V.J., Johnston S.C. (2015). Guidelines for the Management of Patients with Unruptured Intracranial Aneurysms a Guideline for Healthcare Professionals from the American Heart Association/American Stroke Association. Stroke.

[33] Connolly E.S., Rabinstein A.A., Carhuapoma J.R., Derdeyn C., Dion J., Higashida R.T., Hoh B.L., Kirkness C.J., Naidech A.M., Ogilvy C.S. (2012). Guidelines for the Management of Aneurysmal Subarachnoid Hemorrhage A Guideline for Healthcare Professionals from the American Heart Association/American Stroke Association. Stroke.

[34] Etminan N., Aguiar de Sousa D., Tiseo C., Bourcier R., Desal H., Lindgren A., Koivisto T., Netuka D., Peschillo S., Lémeret S. (2022). European Stroke Organisation (ESO) guidelines on management of unruptured intracranial aneurysms. Eur. J. Stroke.

[35] Dandurand C., Zhou L., Prakash S., Redekop G., Gooderham P., Haw C.S. (2021). Cost-effectiveness analysis in patients with an unruptured cerebral aneurysm treated with observation or surgery. J. Neurosurg..

[36] Zhang X., Li L., Hong B., Xu Y., Liu Y., Huang Q., Liu J. (2018). A systematic review and meta-analysis on economic comparison between endovascular coiling versus neurosurgical clipping for ruptured intracranial aneurysms. World Neurosurg..

